# Anti‐epidermal growth factor receptor monoclonal antibody plus palliative chemotherapy as a first‐line treatment for recurrent or metastatic nasopharyngeal carcinoma

**DOI:** 10.1002/cam4.2838

**Published:** 2020-01-19

**Authors:** Chen Chen, Yixin Zhou, Xuanye Zhang, Sha Fu, Zuan Lin, Wenfeng Fang, Yunpeng Yang, Yan Huang, Hongyun Zhao, Shaodong Hong, Li Zhang

**Affiliations:** ^1^ Department of Radiation Oncology Sun Yat‐sen University Cancer Center Guangzhou China; ^2^ State Key Laboratory of Oncology in South China Collaborative Innovation Center for Cancer Medicine Guangdong Key Laboratory of Nasopharyngeal Carcinoma Diagnosis and Therapy Sun Yat‐sen University Cancer Center Guangzhou China; ^3^ Department of VIP region Sun Yat‐sen University Cancer Center Guangzhou China; ^4^ Department of Medical Oncology Sun Yat‐sen University Cancer Center Guangzhou China; ^5^ Guangdong Provincial Key Laboratory of Malignant Tumor Epigenetics and Gene Regulation Pathology Department Sun Yat‐Sen Memorial Hospital Sun Yat‐Sen University Guangzhou China; ^6^ Department of Clinical Research Sun Yat‐sen University Cancer Center Guangzhou China

**Keywords:** anti‐epidermal growth factor receptor, first‐line treatment, monoclonal antibody, palliative chemotherapy, recurrent or metastatic nasopharyngeal carcinoma

## Abstract

**Background:**

Platinum‐based chemotherapy is the standard of care as first‐line treatment for recurrent or metastatic nasopharyngeal carcinoma (RM‐NPC); however, the prognosis of patients with RM‐NPC remains poor. The aim of this study was to evaluate the role of anti‐epidermal growth factor receptor (anti‐EGFR) antibody plus chemotherapy for RM‐NPC.

**Methods:**

RM‐NPC patients who received first‐line chemotherapy plus an anti‐EGFR antibody were recruited from Sun Yat‐Sen University Cancer Center between July 2007 and November 2017. Survival analyses were performed using the Kaplan‐Meier method with a log‐rank test. A Cox proportional hazards model was used for the multivariate analyses.

**Results:**

A total of 203 patients were enrolled in the present study. The median follow‐up time was 34.3 months (interquartile range: 19.7‐66.5 months). The median progression‐free survival (PFS) was 8.9 months (95% CI: 7.7‐10.0 months) and the median overall survival (OS) was 29.1 months (95% CI: 23.5‐34.6 months). The 1‐, 3‐, and 5‐year PFS and OS rates were 35.5% and 79.6%, 15.2% and 42.5%, and 11.6% and 23.6%, respectively. The objective response rate (ORR) was 67.5% and the disease control rate (DCR) was 91.1%. The multivariate analysis identified the following prognostic factors for PFS: anti‐EGFR agent (*P* = .010), recurrence/metastasis sequence (*P* = .016), KPS (*P* = .017), and combined chemotherapy regimen (*P* = .015). Independent risk factors for OS included age >43 years (*P* = .002), Karnofsky performance score ≤80 (*P* < .001), and higher level of baseline Epstein‐Barr virus (EBV) DNA (*P* = .008). Leukopenia was the most common adverse event (AE) in this cohort (any grade, 84.2%; grades 3‐4, 43.4%).

**Conclusions:**

Anti‐EGFR antibody plus chemotherapy achieved promising antitumor activity with a tolerable toxicity profile in RM‐NPC. Thus, randomized clinical trials are warranted to compare the efficacy of chemotherapy with or without anti‐EGFR antibody in these patients.

## Graphical abstract

Anti‐EGFR monoclonal antibody plus chemotherapy achieved promising response rate, progression‐free survival, and overall survival with a tolerable toxicity profile for recurrent or metastatic nasopharyngeal carcinoma.

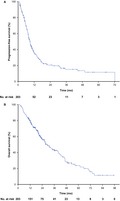

## 1 INTRODUCTION

Nasopharyngeal carcinoma (NPC) is an endemic tumor in the eastern and southeastern regions of Asia. Radiotherapy with or without concurrent chemotherapy is the standard of care for patients with early or locally advanced stage disease. With the development of radiation techniques and systemic treatment, the overall survival (OS) of NPC has improved in recent decades.^1^ However, most patients eventually develop locoregional recurrence and/or distant metastasis.^2^, ^3^, ^4^ For those with recurrence or metastatic NPC (RM‐NPC), the prognosis remains extremely poor, with a median OS ranging from 15 to 29 months.^5^ Therefore, new systemic treatment strategies are urgently required to optimize clinical outcomes.

Epidermal growth factor receptor (EGFR) is a transmembrane glycoprotein and a member of the erbB family of tyrosine kinase receptors. In addition, EGFR is involved in the regulation of cellular proliferation, differentiation, and survival. Thus, EGFR represents one of the most attractive targets for cancer therapy due to the fact that many solid tumors, including NPC, display EGFR overexpression.^6^, ^7^ In particular, EGFR overexpression is observed in more than 90% of NPC and is associated with a poor prognosis.^8^, ^9^ Moreover, anti‐EGFR monoclonal antibodies (mAbs) were found to inhibit the activation of EGFR downstream signaling pathways by blocking its extracellular association with its ligands.^10^ Therefore, anti‐EGFR mAbs are considered to be a promising agent for NPC.

Cetuximab (CTX) and nimotuzumab (NTZ) are two major anti‐EGFR mAbs that have frequently been used for the treatment of NPC. Several studies have indicated that treatment with CTX or NTZ enhances the efficacy of chemoradiotherapy for locoregionally advanced NPC.^11^, ^12^, ^13^, ^14^, ^15^ However, the antitumor activity of anti‐EGFR mAbs for RM‐NPC has rarely been reported.^16^, ^17^ The aim of the present study was to evaluate the antitumor efficacy and safety of anti‐EGFR mAbs (CTX or NTZ) plus palliative chemotherapy as a first‐line treatment for RM‐NPC.

## 2 MATERIALS AND METHODS

### 2.1 Study population

**Figure 1:**
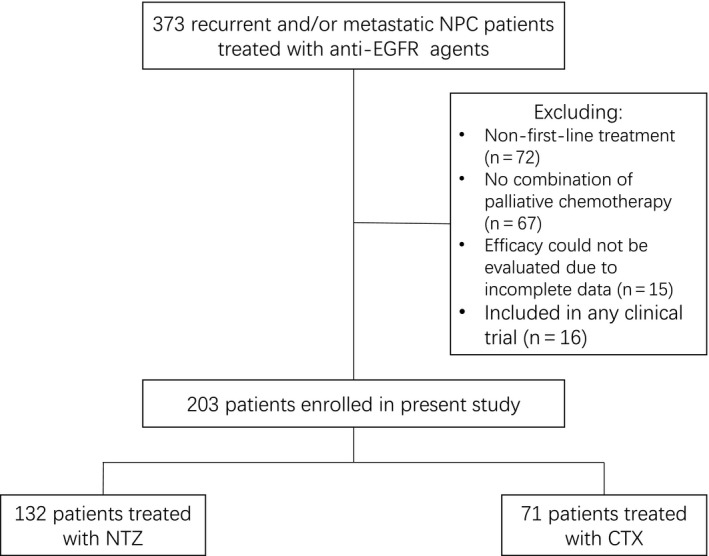
Diagram illustrating the patient selection process. NPC, nasopharyngeal carcinoma; EGFR, anti‐epidermal growth factor receptor; NTZ, nimotuzumab; CTX, cetuximab

We recruited consecutive patients from Sun Yat‐Sen University Cancer Center between July 2007 and November 2017, who met the following criteria: (a) histologically confirmed NPC; (b) metastatic or recurrent disease following primary standard treatment, or primarily metastasis; (c) no history of previous systemic chemotherapy for recurrent or metastatic disease; (d) received palliative chemotherapy plus anti‐EGFR mAbs; (e) received at least one cycle of anti‐EGFR mAbs; (f) ability to be evaluated with complete clinical data; and (g) received study treatment outside of anti‐EGFR therapy clinical trials as indicated by the medical records. Figure 1 illustrates the process of patient selection.

The institutional review board of Sun Yat‐sen University Cancer Center approved this retrospective study and waived the need for informed consent (written or verbal).

### 2.2 Data collection

The collected data contained: (a) gender, age, smoking status, Karnofsky performance score (KPS),^18^ and Epstein‐Barr virus (EBV) DNA count before the administration of anti‐EGFR agents; (b) pathological histology, recurrence/metastasis sequence (synchronous or metachronous with respect to the primary diagnosis of NPC); (c) type of anti‐EGFR agent (NTZ or CTX) combined chemotherapy regimens; (d) imaging information for the evaluation of treatment efficacy; and (e) survival status and time point.

The plasma EBV DNA concentration prior to treatment with the anti‐EGFR agent was measured using a real‐time quantitative polymerase chain reaction as previously described by our institution.^19^ According to the EBV DNA concentration, four groups were defined by magnitudes of 10 and another group was established for unknown levels. All adverse events (AEs) were graded according to the National Cancer Institute Common Toxicity Criteria for Adverse Events version 4.0.

### 2.3 Treatment

All patients received palliative chemotherapy plus an anti‐EGFR agent as first‐line treatment. Palliative chemotherapy included four common regimens: (a) taxane plus cisplatin/nedaplatin/carboplatin and fluorouracil (TPF); (b) taxane plus cisplatin/nedaplatin/carboplatin (TP); (c) fluorouracil plus cisplatin/nedaplatin/carboplatin (PF); and (d) gemcitabine plus cisplatin/nedaplatin/carboplatin (GP). Anti‐EGFR agents included the intravenous administration of NTZ or CTX prior to chemotherapy. Given the retrospective nature of this study, the treatment regimens were directly extracted from the electronic patient records. Treatment decisions were made according to the discretion of the treating physicians and the patients' desire, which were considered to be based on factors including, but not limited to, the patient's economic situation, complications, the patient's physical condition, and the doctor's preference. The dosage, administration, and modification of these drugs were determined according to the locally approved formulary information of the treating physicians.

### 2.4 Endpoints and statistical analysis

The primary endpoint was progression‐free survival (PFS), which was defined as the time from the initiation of first‐line therapy to the date of disease progression or death from any cause, whichever came first. Secondary endpoints included OS and the tumor response. OS was defined as the time from the beginning of first‐line therapy to the date of death due to any cause. The tumor response was assessed by regular imaging per RECIST version 1.1, which consisted of complete response (CR), partial response (PR), stable disease (SD), and progressive disease (PD). All medical imaging was independently reviewed by the first and second authors of this study. In case of any discrepancies, the final decision was made by a full discussion involving the corresponding author(s). The objective response rate (ORR) was defined as the proportion of patients achieving a complete or partial response, and disease control was defined as the proportion of CR + PR + SD. To explore the prognostic factors, interventions, including anti‐EGFR mAbs and chemotherapy regimens, were included in the multivariate analysis as important confounding factors together with baseline characteristics.

A Chi‐square test was used to distinguish the distributional differences of the categorical variables. The Kaplan‐Meier method with a log‐rank test was used to calculate and compare the cumulative survival rates. A Cox proportional hazards model was used for the multivariate analysis and to estimate the hazard ratio (HR) with 95% confidence intervals (CIs). Statistical analyses were performed using SPSS version 21.0 (Chicago, IL, USA). A threshold two‐sided *P* value of less than 0.05 was considered significant.

## 3 RESULTS

### 3.1 Patient demographic characteristics

A total of 373 RM‐NPC patients treated with anti‐EGFR agents were screened and 203 patients were finally included in this study. The baseline characteristics of the total patients are listed in Table 1 and the baseline characteristics of the patients in each of the different chemotherapy regimens are listed in Table S1. The median age was 43 years (range: 12‐72 years). The primary pathological histology consisted of undifferentiated non‐keratinized carcinoma (n = 187, 92.1%). Other types of pathological histology consisted of non‐keratosis (n = 3, 1.5%), differentiated non‐keratosis (n = 6, 3.0%), squamous carcinoma (n = 3, 1.5%), and unknown type (n = 4, 2.0%). A total of 100 (49.3%) patients were initially diagnosed with distant metastases (synchronous metastasis), and 103 (50.7%) patients experienced recurrence or metastasis secondary to the initial treatment (metachronous metastasis). A total of 132 (65.0%) patients received NTZ, and 71 (35.0%) patients received CTX. More patients received TP (n = 84, 41.4%) as a combined chemotherapy regimen.

**Table 1:** The baseline characteristics of patients

Characters	Patients (%)
Gender	
Male	168 (82.8)
Female	35 (17.2)
Age	
≤43 y	98 (48.3)
>43 y	105 (51.7)
Smoke	
Yes	65 (32.0)
No	138 (68.0)
Anti‐EGFR agent	
Nimotuzumab	132 (65.0)
Cetuximab	71 (35.0)
Pathological histology	
Undifferentiated non‐keratosis	187 (92.0)
Others†	16 (8.0)
Recurrence/Metastasis sequence	
Synchronous	100 (49.3)
Metachronous	103 (50.7)
Karnofsky Performance Score (KPS)	
>80	173 (85.2)
≤80	30 (14.8)
Baseline Epstein‐Barr virus DNA level (copies/mL)	
<10E3	26 (12.8)
≥10E3 and < 10E4	32 (15.8)
≥10E4 and < 10E5	57 (28.1)
≥10E5	67 (33.0)
Unknown	21 (10.3)
Combined chemotherapy regimen	
TPF	47 (23.2)
TP	84 (41.4)
PF	24 (11.8)
GP	37 (18.2)
Others‡	11 (5.4)

Abbreviations: EGRF, epidermal growth factor receptor; TPF, taxane plus cisplatin/nedaplatin/carboplatin and fluorouracil; TP, taxane plus cisplatin/nedaplatin/carboplatin; PF, fluorouracil plus cisplatin/nedaplatin/carboplatin; GP, gemcitabine plus cisplatin/nedaplatin/carboplatin.

^†^ Other pathological histology types contained non‐keratosis, differentiated non‐keratosis, squamous carcinoma, and unknown type.

^‡^ Other chemotherapy regimens included pemetrexed + cisplatin/nedaplatin, pemetrexed + gemcitabine, gemcitabine + capecitabine/S‐1, gemcitabine + oxaliplatin, and gemcitabine + vincristine.

### 3.2 Survival analysis and tumor response

**Figure 2:**
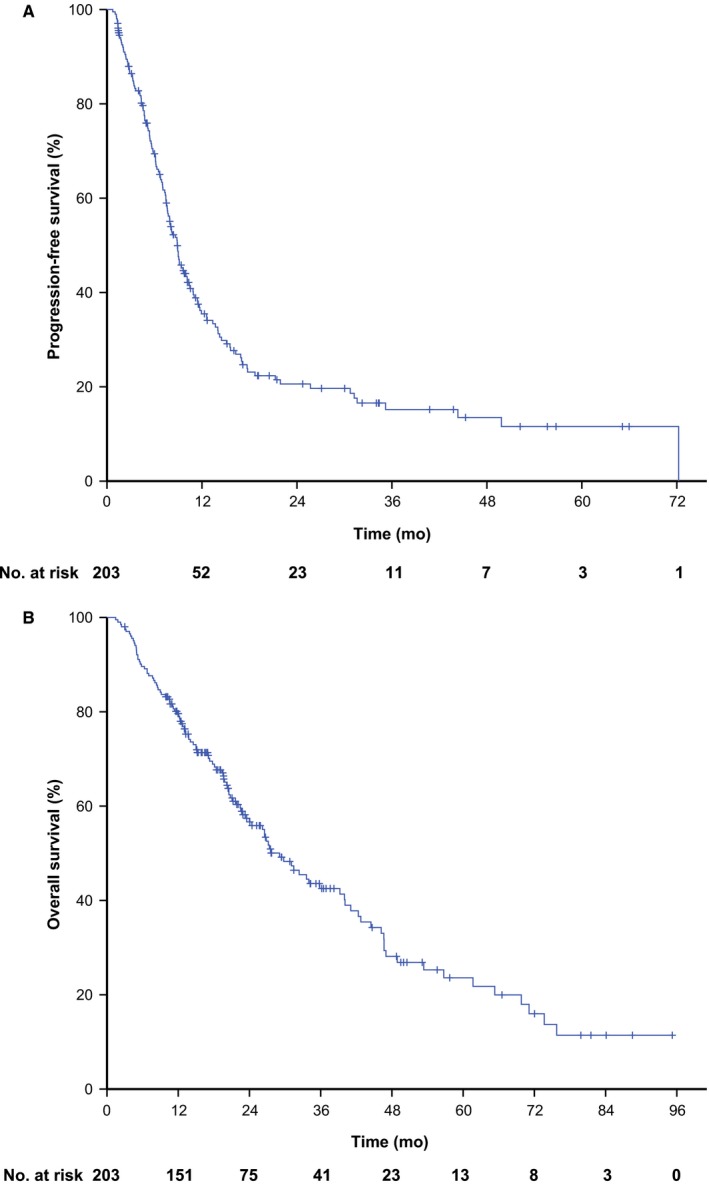
Survival curves of recurrent or metastatic nasopharyngeal carcinoma treated with anti‐epidermal growth factor receptor monoclonal antibody plus palliative chemotherapy as first‐line therapy. A, progression‐free survival curve; B, overall survival curve

The cutoff date for the data was 31 October 2018. The median follow‐up time was 34.3 months (interquartile range: 19.7‐66.5 months). During the follow‐up period, 145 (71.4%) patients displayed progressive disease and 115 (56.7%) patients died after undergoing first‐line palliative treatment (survival curves are shown in Figure 2). The 1‐, 3‐, and 5‐year PFS rates were 35.5%, 15.2%, and 11.6%, respectively. In contrast, the 1‐, 3‐, and 5‐year OS rates were 79.6%, 42.5%, and 23.6%, respectively.

The median PFS was 8.9 months (95% CI: 7.7‐10.0 months) and the median OS was 29.1 months (95% CI: 23.5‐34.6 months). Specifically, the median PFS and OS of the patients with different characteristic are listed in Table 2. Moreover, patients with a higher level of EBV DNA exhibited a longer median survival time. Among the 203 patients, eight (3.9%) achieved CR, 129 (63.6%) had PR, 48 (23.6%) had SD, and 18 (8.9%) had PD as the best response, respectively. The ORR was 67.5%, and the disease control rate (DCR) was 91.1%.

**Table 2:** Univariate and multivariate analyses of progression‐free survival and overall survival

characters	Progression‐free survival				Overall survival			
	Median months (95% CI)	*P*‐uni	*P*‐multi	HR (95% CI)	Median months (95% CI)	*P*‐uni	*P*‐multi	HR (95% CI)
Gender								
Male	8.9 (7.7‐10.2)	.80	.81	1	29.8 (23.8‐35.8)	.74	.21	1
Female	8.9 (6.7‐11.1)			1.058 (0.663‐1.689)	26.5 (6.8‐46.2)			1.384 (0.838‐2.285)
Age								
≤43 y	10.0 (8.4‐11.5)	.071	.060	1	34.0 (20.6‐47.4)	.096	.002	1
>43 y	8.0 (6.8‐9.2)			1.398 (0.986‐1.984)	27.2 (19.2‐35.2)			1.861 (1.248‐2.775)
Smoke								
Yes	8.6 (7.2‐10.1)	.61	NA	NA	29.1 (19.5‐38.7)	.62	NA	NA
No	9.0 (7.3‐10.7)				27.4 (19.4‐35.3)			
Anti‐EGFR agent								
NTZ	7.9 (6.1‐9.7)	.054	.010	1	27.2 (18.6‐35.7)	.21	.15	1
CTX	9.7 (7.7‐11.7)			0.623 (0.435‐0.891)	32.4 (19.6‐45.1)			0.736 (0.485‐1.118)
Pathological histology								
Undifferentiated non‐keratosis	8.9 (7.8‐9.9)	.62	NA	NA	27.6 (21.8‐33.3)	.51	NA	NA
Others†	9.7 (2.0‐17.4)				34.8 (0.0‐114.8)			
Recurrence/Metastasis sequence								
Synchronous	10.0 (8.2‐11.7)	.039	.016	1	31.1 (23.3‐38.9)	.33	NA	NA
Metachronous	7.9 (6.8‐9.0)			1.629 (1.094‐2.424)	26.8 (17.7‐36.0)			
Karnofsky Performance Score (KPS)								
>80	9.1 (7.7‐10.4)	.007	.017	1	33.6 (22.4‐44.8)	＜.001	＜.001	1
≤80	5.4 (3.0‐7.7)			1.803 (1.114‐2.919)	11.8 (1.2‐22.4)			2.749 (1.682‐4.496)
Baseline Epstein‐Barr virus DNA level (copies/mL)								
<10E3	15.5 (6.7‐24.4)	.051	.26	1	61.7 (32.3‐91.0)	.009	.008	1
≥10E3 and <10E4	10.9 (7.9‐13.8)			1.420 (0.723‐2.790)	46.7 (37.5‐55.8)			1.715 (0.739‐3.979)
≥10E4 and <10E5	8.2 (6.6‐9.9)			1.709 (0.917‐3.184)	26.5 (19.9‐33.2)			2.285 (1.098‐4.756)
≥10E5	7.3 (5.5‐9.1)			1.919 (1.041‐3.536)	20.5 (16.8‐24.3)			3.445 (1.684‐7.047)
Unknown	12.6 (0.0‐29.3)			1.244 (0.589‐2.629)	26.2 (4.7‐47.7)			2.944 (1.329‐6.519)
Combined chemotherapy regimen								
TPF	9.7 (6.5‐12.9)	.082	.015	1	40.0 (28.4‐51.6)	.16	.082	1
TP	8.2 (6.4‐9.9)			1.896 (1.212‐2.966)	24.0 (18.3‐29.7)			1.915 (1.145‐3.203)
PF	6.6 (5.8‐7.5)			1.636 (0.866‐3.093)	21.7 (8.9‐34.5)			2.037 (1.100‐3.775)
GP	12.6 (7.1‐18.1)			0.935 (0.511‐1.709)	31.5 (22.5‐40.5)			1.652 (0.860‐3.175)
Others‡	7.7 (5.8‐9.5)			1.542 (0.737‐3.226)	48.9 (6.1‐91.7)			0.987 (0.374‐2.605)

Abbreviations: NTZ, Nimotuzumab; CI, confidence interval; CTX, Cetuximab; GP, gemcitabine plus cisplatin/nedaplatin/carboplatin; HR, hazard ratio; PF, fluorouracil plus cisplatin/nedaplatin/carboplatin; *P*‐uni, *P* value for univariate analysis; *P*‐multi, *P* value for multivariate analysis; TP, taxane plus cisplatin/nedaplatin/carboplatin; TPF, taxane plus cisplatin/nedaplatin/carboplatin and fluorouracil.

^†^ Other pathological histology types contained non‐keratosis, differentiated non‐keratosis, squamous carcinoma, and unknown type.

^‡^ Other chemotherapy regimens included pemetrexed + cisplatin/nedaplatin, pemetrexed + gemcitabine, gemcitabine + capecitabine/S‐1, gemcitabine + oxaliplatin, and gemcitabine + vincristine.

### 3.3 Prognostic analysis

**Figure 3:**
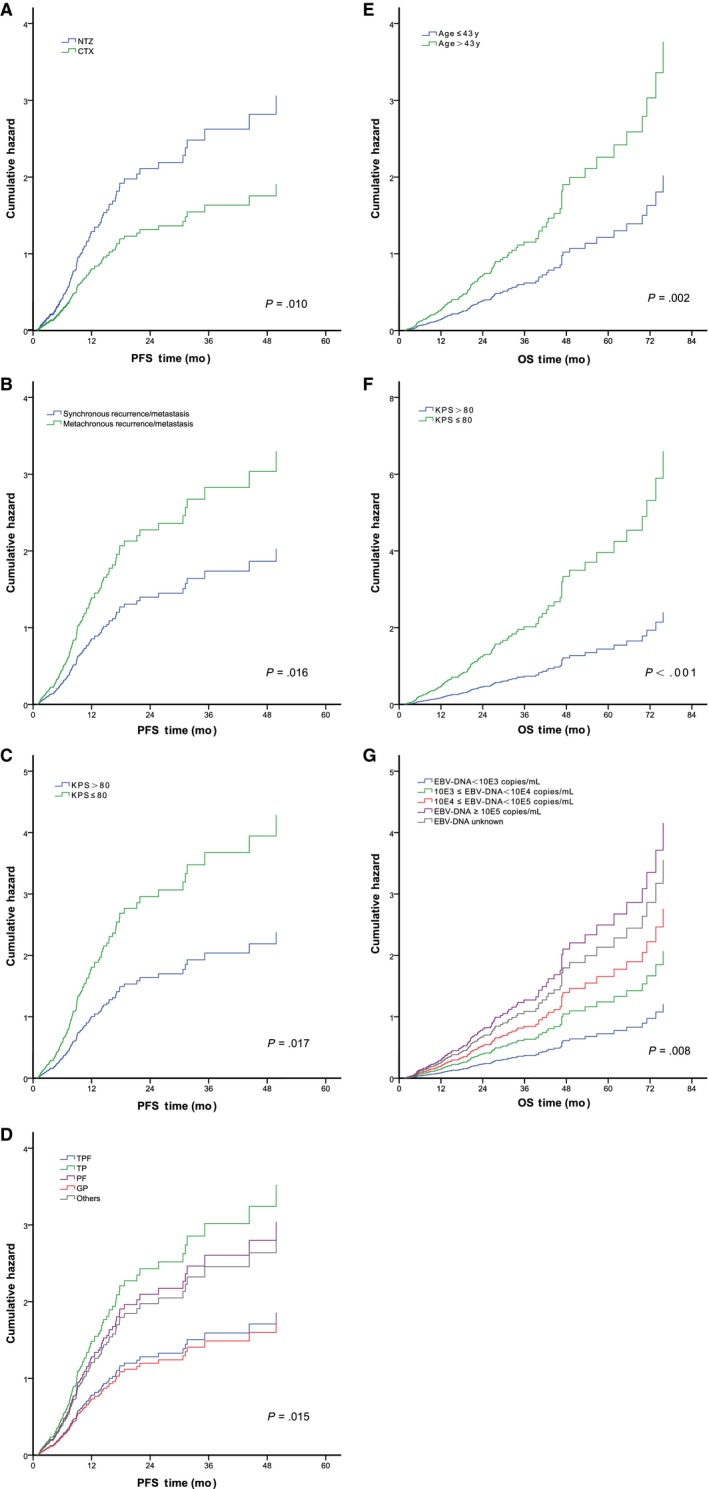
Cumulative hazard curves of the independent risk factors identified by multivariate analyses for progression‐free survival and overall survival, respectively. A, type of anti‐EGFR agent for PFS; B, recurrence/metastasis sequence for PFS; C, KPS for PFS; D, chemotherapy regimen for PFS; E, age for OS; F, KPS for OS; and G, EBV DNA level for OS. EGFR, anti‐epidermal growth factor receptor; PFS, progression‐free survival; OS, overall survival; KPS, Karnofsky performance score; EBV, Epstein‐Barr virus; NTZ, Nimotuzumab; CTX, Cetuximab; TPF, taxane plus cisplatin/nedaplatin/carboplatin and fluorouracil; TP, taxane plus cisplatin/nedaplatin/carboplatin; PF, fluorouracil plus cisplatin/nedaplatin/carboplatin; GP, gemcitabine plus cisplatin/nedaplatin/carboplatin; Other chemotherapy regimens included pemetrexed + cisplatin/nedaplatin, pemetrexed + gemcitabine, gemcitabine + capecitabine/S‐1, gemcitabine + oxaliplatin, and gemcitabine + vincristine

Univariate and multivariate analyses of the PFS and OS are presented in Table 2. The univariate analysis revealed that recurrence/metastasis sequence and KPS had a significant effect on the PFS. The anti‐EGFR agent (*P* = .054) and baseline level of EBV DNA (*P* = .051) were associated with a potential effect. The multivariate analysis identified four independent prognostic factors for PFS, including the anti‐EGFR agent (*P* = .010), recurrence/metastasis sequence (*P* = .016), KPS (*P* = .017), and combined chemotherapy regimen (*P* = .015) (corresponding hazard ratios are listed in Table 2, cumulative hazard curves are shown in Figure 3 A‐D). Age (*P* = .060) was a potential prognostic factor.

For the OS, the multivariate analysis confirmed that an older age (age > 43 years) (*P* = .002), poor KPS (KPS ≤ 80) (*P* < .001), and higher level of baseline EBV DNA (*P* = .008) were independent risk factors (Table 2; cumulative hazard curves are shown in Figure 3E‐G). A combined chemotherapy regimen was a potential prognostic factor (*P* = .082).

### 3.4 Toxicity analysis

Common treatment‐related AEs are summarized in Table 3. A total of 192 patients (94.6%) experienced at least one AE, among whom 121 patients were treated with NTZ (121/132, 91.7%) and 71 patients were treated with CTX (71/71, 100%). The most common AE was leukopenia (n = 171, 84.2%) followed by decreased appetite (n = 135, 66.5%) and nausea (n = 123, 60.6%). With the exception of severe hematologic toxicity, including grades 3‐4 leukopenia (n = 88, 43.4%) and thrombocytopenia (n = 23, 11.3%), other grades 3‐4 AEs were rare (occurrence rate < 5%). Compared with patients who received NTZ, the patients treated with CTX were more likely to suffer from mucosal inflammation (CTX vs NTZ: 31.0% vs 13.6%), weight loss (CTX vs NTZ: 42.3% vs 23.5%), rash (CTX vs NTZ: 33.8% vs 2.3%), fever (CTX vs NTZ: 40.9% vs 23.5%), ALT elevation (CTX vs NTZ: 53.5% vs 29.5%), and AST elevation (CTX vs NTZ: 45.1% vs 24.2%). The cases of grades 3‐4 toxicity were comparable between the patients treated with CTX and NTZ, except for rash (CTX vs NTZ: 4.2% vs 0.0%). For the chemotherapy regimens, patients treated with GP were more likely to have thrombocytopenia (75.7%), including grades 3‐4 thrombocytopenia (32.4%), compared to other regimens (detailed in Table S2).

**Table 3:** Common treatment‐related adverse events

Adverse events	No (%)	Grade 1 (%)	Grade 2 (%)	Grade 3 (%)	Grade 4 (%)	Grade 3 + 4 (%)	All grade (%)
Leukopenia							
Total	32 (15.8)	21 (10.3)	62 (30.5)	69 (34.0)	19 (9.4)	88 (43.4)	171 (84.2)
NTZ group	25 (18.9)	13 (9.9)	45 (34.1)	38 (28.8)	11 (8.3)	49 (37.1)	107 (81.1)
CTX group	7 (9.8)	8 (11.3)	17 (23.9)	31 (43.7)	8 (11.3)	39 (55.0)	64 (90.2)
Thrombocytopenia							
Total	120 (59.1)	29 (14.3)	31 (15.3)	13 (6.4)	10 (4.9)	23 (11.3)	83 (40.9)
NTZ group	80 (60.6)	17 (12.9)	19 (14.4)	10 (7.6)	6 (4.5)	16 (12.1)	52 (39.4)
CTX group	40 (56.4)	12 (16.9)	12 (16.9)	3 (4.2)	4 (5.6)	7 (9.8)	31 (43.6)
Vomiting							
Total	124 (61.1)	68 (33.5)	9 (4.4)	2 (1.0)	0 (0.0)	2 (1.0)	79 (38.9)
NTZ group	83 (62.9)	45 (34.1)	2 (1.5)	2 (1.5)	0 (0.0)	2 (1.5)	49 (37.1)
CTX group	41 (57.7)	23 (32.4)	7 (9.9)	0 (0.0)	0 (0.0)	0 (0.0)	30 (42.3)
Nausea							
Total	80 (39.4)	101 (49.7)	20 (9.9)	2 (1.0)	0 (0.0)	2 (1.0)	123 (60.6)
NTZ group	52 (39.4)	65 (49.3)	13 (9.8)	2 (1.5)	0 (0.0)	2 (1.5)	80 (60.6)
CTX group	28 (39.4)	36 (50.7)	7 (9.9)	0 (0.0)	0 (0.0)	0 (0.0)	43 (60.6)
Mucosal inflammation							
Total	163 (80.3)	24 (11.8)	14 (6.9)	2 (1.0)	0 (0.0)	2 (1.0)	40 (19.7)
NTZ group	114 (86.4)	11 (8.3)	6 (4.5)	1 (0.8)	0 (0.0)	1 (0.8)	18 (13.6)
CTX group	49 (69.0)	13 (18.3)	8 (11.3)	1 (1.4)	0 (0.0)	1 (1.4)	22 (31.0)
Decreased appetite							
Total	68 (33.5)	120 (59.1)	15 (7.4)	0 (0.0)	0 (0.0)	0 (0.0)	135 (66.5)
NTZ group	48 (36.3)	74 (56.1)	10 (7.6)	0 (0.0)	0 (0.0)	0 (0.0)	84 (63.7)
CTX group	20 (28.2)	46 (64.8)	5 (7.0)	0 (0.0)	0 (0.0)	0 (0.0)	51 (71.8)
Diarrhea							
Total	175 (86.2)	24 (11.8)	2 (1.0)	2 (1.0)	0 (0.0)	2 (1.0)	28 (13.8)
NTZ group	116 (87.8)	14 (10.6)	1 (0.8)	1 (0.8)	0 (0.0)	1 (0.8)	16 (12.2)
CTX group	59 (83.1)	10 (14.1)	1 (1.4)	1 (1.4)	0 (0.0)	1 (1.4)	12 (16.9)
Nephrotoxicity							
Total	163 (80.3)	38 (18.7)	2 (1.0)	0 (0.0)	0 (0.0)	0 (0.0)	40 (19.7)
NTZ group	102 (77.3)	29 (21.9)	1 (0.8)	0 (0.0)	0 (0.0)	0 (0.0)	30 (22.7)
CTX group	61 (85.9)	9 (12.7)	1 (1.4)	0 (0.0)	0 (0.0)	0 (0.0)	10 (14.1)
Hypotension							
Total	169 (83.3)	34 (16.7)	0 (0.0)	0 (0.0)	0 (0.0)	0 (0.0)	34 (16.7)
NTZ group	114 (86.4)	18 (13.6)	0 (0.0)	0 (0.0)	0 (0.0)	0 (0.0)	18 (13.6)
CTX group	55 (77.5)	16 (22.5)	0 (0.0)	0 (0.0)	0 (0.0)	0 (0.0)	16 (22.5)
Weight loss							
Total	142 (70.0)	45 (22.1)	16 (7.9)	0 (0.0)	0 (0.0)	0 (0.0)	61 (30.0)
NTZ group	101 (76.5)	23 (17.4)	8 (6.1)	0 (0.0)	0 (0.0)	0 (0.0)	31 (23.5)
CTX group	41 (57.7)	22 (31.0)	8 (11.3)	0 (0.0)	0 (0.0)	0 (0.0)	30 (42.3)
Rash							
Total	176 (86.6)	20 (9.9)	4 (2.0)	3 (1.5)	0 (0.0)	3 (1.5)	27 (13.4)
NTZ group	129 (97.7)	3 (2.3)	0 (0.0)	0 (0.0)	0 (0.0)	0 (0.0)	3 (2.3)
CTX group	47 (66.2)	17 (24.0)	4 (5.6)	3 (4.2)	0 (0.0)	3 (4.2)	24 (33.8)
Fever							
Total	143 (70.4)	48 (23.7)	12 (5.9)	0 (0.0)	0 (0.0)	0 (0.0)	60 (29.6)
NTZ group	101 (76.5)	27 (20.5)	4 (3.0)	0 (0.0)	0 (0.0)	0 (0.0)	31 (23.5)
CTX group	42 (59.1)	21 (29.6)	8 (11.3)	0 (0.0)	0 (0.0)	0 (0.0)	29 (40.9)
ALT elevation							
Total	126 (62.1)	62 (30.5)	8 (3.9)	7 (3.5)	0 (0.0)	7 (3.5)	77 (37.9)
NTZ group	93 (70.5)	33 (25.0)	2 (1.5)	4 (3.0)	0 (0.0)	4 (3.0)	39 (29.5)
CTX group	33 (46.5)	29 (40.8)	6 (8.5)	3 (4.2)	0 (0.0)	3 (4.2)	38 (53.5)
AST elevation							
Total	139 (68.4)	54 (26.6)	5 (2.5)	5 (2.5)	0 (0.0)	5 (2.5)	64 (31.6)
NTZ group	100 (75.8)	25 (18.9)	4 (3.0)	3 (2.3)	0 (0.0)	3 (2.3)	32 (24.2)
CTX group	39 (54.9)	29 (40.9)	1 (1.4)	2 (2.8)	0 (0.0)	2 (2.8)	32 (45.1)

Abbreviations: ALT, alanine aminotransferase; AST, aspartate aminotransferase; CTX, cetuximab; NTZ, nimotuzumab.

## 4 DISCUSSION

Patients with RM‐NPC have very poor survival outcomes due to therapeutic resistance. Therefore, novel treatment strategies are required to optimize clinical outcomes. To our knowledge, this is the largest cohort study that has evaluated the antitumor activity of an anti‐EGFR mAb plus chemotherapy as a first‐line treatment for RM‐NPC. Our findings indicate that anti‐EGFR mAbs plus chemotherapy achieved a promising response rate, PFS, and OS for RM‐NPC.

The first head‐to‐head randomized phase III clinical trial conducted by our group^20^ established the standard first‐line chemotherapy regimen for RM‐NPC based on the fact that GP achieved a longer PFS than PF (median: 7.0 vs 5.6 months, respectively). The response rates for GP and PF were 64% and 42% (*P* < .001), respectively. While these findings were statistically significant, clinically mild improvements indicate that the efficacy of chemotherapy alone has reached a plateau. It has been recognized that patients with RM‐NPC are likely to harbor platinum‐resistant tumor clones, which may be partially due to the activation of the EGFR signaling pathway.^21^, ^22^ Therefore, it is rational that blocking the EGFR pathway could resensitize these tumor clones to chemotherapy and delay disease progression.^6^, ^8^, ^9^ Moreover, Chan et al^17^ conducted a phase II trial, which included 60 RM‐NPC patients who had been heavily treated with platinum‐based chemotherapy for recurrent or metastatic disease. These patients were treated with cetuximab plus carboplatin, which yielded a median PFS of 81 days, ORR of 11.7%, and DCR of 60%. Recently, Zhao et al^16^ conducted a single arm, phase II study to evaluate the role of NTZ plus PF as a first‐line treatment for 35 patients with RM‐NPC. The median PFS was 7.0 months, median OS was 16.3 months, and ORR was 71.4%. However, since both trials enrolled a very limited number of patients, there is limited confidence in the data interpretation. The efficacy of treatment with anti‐EGFR mAbs plus chemotherapy must be validated in a larger population. Therefore, we conducted this retrospective study with 203 RM‐NPC patients treated with either CTX or NTZ plus palliative chemotherapy as first‐line therapy. The median PFS was 8.9 months (95% CI: 7.7‐10.0 months) and the median OS was 29.1 months (95% CI: 23.5‐34.6 months). The ORR and DCR were 67.5% and 91.1%, respectively. The survival outcomes of the above mentioned studies and our study are summarized in Table 4.

**Table 4:** Survival outcomes comparison between other previous studies and our present study

Author	Year of case	Patients number	Arms/Cohort	Therapeutic regimen	PFS	OS	ORR	DCR
Zhang^20^	2012‐2015	362	Arm 1	GP	7.0 mo	29.1 mo	64%	90%
			Arm 2	PF	5.6 mo	20.9 mo	42%	86%
Chan^17^	Published in 2005	60	Single arm	CTX + Carboplatin	81 d	NA	11.2%	61%
Zhao^16^	2012‐2015	35	Single arm	NTZ + PF	7.0 mo	16.3 mo	71.4%	85.7%
Present study	2007‐2017	203	Whole cohort	CTX/NTZ + palliative chemotherapy	8.9 mo	29.1 mo	67.5%	91.1%

Abbreviations: CTX, Cetuximab; DCR, disease control rate; GP, gemcitabine plus cisplatin; NA, not applicable; NTZ, Nimotuzumab; OS, overall survival; ORR, objective response rate; PF, fluorouracil plus cisplatin; PFS, progression‐free survival.

Using univariate and multivariate analyses, several prognostic factors were confirmed for the PFS and OS, respectively. In addition, NTZ treatment, metachronous metastasis, and poor KPS were found to be independent risk factors for PFS. CTX was associated with a longer PFS than those with NTZ, but not for OS, which may be due to the impact of post‐progression treatment. Therefore, head‐to‐head randomized studies are required to confirm whether there is a real difference regarding the efficacy of CTX and NTZ. The poor PFS of metachronous metastasis compared to synchronous metastasis may be attributed to greater therapeutic resistance.^23^, ^24^, ^25^ We also found that while the combined chemotherapy regimen was an independent prognostic factor for PFS, the optimal chemotherapy regimens were not determined in the current study. Recently, a meta‐analysis showed that the triple combination regimen was associated with the best short‐term efficacy but failed to improve the patient prognosis among the four commonly used first‐line chemotherapy regimens (PF, GP, TP, and triplet combination regimen) for RM‐NPC.^26^

An age older than 43 years, KPS ≤ 80, and a higher level of baseline EBV DNA were found to be independent risk factors for OS. Patients older than 43‐year‐old had a shorter OS than young patients. Moreover, age was a common influential factor for the outcomes of many metastatic cancers, including breast cancer,^27^ colon cancer,^28^ and NPC patients with bone metastases.^29^ Thus, these findings indicate that older patients may be associated with poor treatment tolerance. In addition, poor KPS was identified as an independent risk factor for OS, even if there was no effect on PFS. This finding is consistent with the results of Zheng et al,^24^ who found that multimodal treatment could improve the survival of metastatic NPC patients who exhibited a good performance status. Patients with favorable KPS had increased opportunity for posterior‐line therapy. As an important prognostic factor for NPC,^1^, ^30^, ^31^ EBV DNA also exhibited a positive correlation with OS risk in patients with RM‐NPC. With an increased level of EBV DNA, the OS risk increased monotonously.

The overall toxicity profile was tolerable in this study. Severe toxicity was primarily associated with hematology, but could be managed by medical intervention. Moderate mucosal inflammation, rash, fever, and liver functional damage were more common in patients treated with CTX than those treated with NTZ, which may be related to the drug properties of these two mAbs. Greater thrombocytopenia was observed in patients with GP, which was primarily attributed to gemcitabine.

Despite these findings, our study has several limitations: (a) since this was a retrospective nonrandomized study, there are potential confounding factors, including the chemotherapy regimens and anti‐EGFR therapies, which may affect the interpretation of the results. However, we attempted to compensate for this deficiency by performing multivariate analyses; (b) the first‐line palliative chemotherapy regimens were variable in this study. We are unable to reveal the most suitable chemotherapy regimens that can be combined; (c) we lacked information regarding EGFR expression in our cohort, which is due to the retrospective nature of this study, technical challenges of the tissue sampling process, and the difficulty of conducting robust interlaboratory quality assurance of EGFR expression; (d) no head‐to‐head comparison of efficacy was made between treatment with anti‐EGFR mAbs plus chemotherapy and chemotherapy alone in this study; and (e) due to the inherent limitations of this retrospective study in terms of AE reporting, we have only listed AE items that could be obtained from the patient medical records and laboratory results.

In conclusion, the anti‐EGFR mAbs (CTX or NTZ) combined with palliative chemotherapy achieved promising antitumor activity with a tolerable toxicity profile as a first‐line treatment for RM‐NPC. Thus, further studies are urgently required to verify our findings and to compare the safety and efficacy of anti‐EGFR mAbs plus chemotherapy with chemotherapy alone for the treatment of RM‐NPC.

## FUNDING AND ACKNOWLEDGMENTS

This study was funded by the Natural Science Foundation of Guangdong Province (No. 2018A030310236); the Outstanding Young Talents Program of Sun Yat‐sen University Cancer Center (16zxyc04); the Central Basic Scientific Research Fund for Colleges‐Young Teacher Training Program of Sun Yat‐sen University (17ykpy81). We sincerely appreciate Yidu Cloud Corporation (Beijing, China) for data retrieval and management.

## CONFLICT OF INTEREST

There are none potential conflict of interest.

## AUTHOR CONTRIBUTIONS

Chen Chen: Conceptualization, data curation, formal analysis, methodology, investigation, writing–original draft, and writing–review and editing. Yixin Zhou: Conceptualization, data curation, formal analysis, methodology, investigation, writing–original draft, and writing–review and editing. Xuanye Zhang: Conceptualization, data curation, formal analysis, methodology, investigation, writing–original draft, and writing–review and editing. Sha Fu: Formal analysis, methodology, and writing–review and editing. Zuan Lin: Methodology and writing–review and editing. Wenfeng Fang: Analysis and writing–review and editing. Yunpeng Yang: Analysis and writing–review and editing. Yan Huang: Analysis and writing–review and editing. Hongyun Zhao: Analysis and writing–review and editing. Shaodong Hong: Conceptualization, data curation, formal analysis, supervision, writing–original draft, and writing–review and editing. Li Zhang: Conceptualization, data curation, formal analysis, supervision, writing–original draft, and writing–review and editing.

## Data Availability

The data that support the findings of this study are available upon request from the corresponding author. The data are not publicly available due to privacy or ethical restrictions.
