# Supplementary material for: Anti‐epidermal growth factor receptor monoclonal antibody plus palliative chemotherapy as a first‐line treatment for recurrent or metastatic nasopharyngeal carcinoma
Source: Cancer Med. 2020 Jan 19;9(5):1721–32. doi: 10.1002/cam4.2838 (PMC7050081; doi:10.1002/cam4.2838)
Supplement: Supplementary file 1 [file CAM4-9-1721-s001.docx]

sTable 1 The baseline characteristics of patients in different chemotherapy regimens

| Characters | Patients (%) | | | | |
| --- | --- | --- | --- | --- | --- |
|  | TPF | TP | PF | GP | Others^‡^ |
| Gender | | | | | |
| Male | 38 (80.9) | 68 (81.0) | 23 (95.8) | 29 (78.4) | 10 (90.9) |
| Female | 9 (19.1) | 16 (19.0) | 1 (4.2) | 8 (21.6) | 1 (9.1) |
| Age | | | | | |
| ≤ 43y | 20 (42.6) | 42 (50.0) | 16 (66.7) | 18 (48.6) | 2 (18.2) |
| ＞ 43y | 27 (57.4) | 42 (50.0) | 8 (33.3) | 19 (51.4) | 9 (81.8) |
| Smoke | | | | | |
| Yes | 17 (36.2) | 25 (29.8) | 10 (41.7) | 9 (24.3) | 4 (36.4) |
| No | 30 (63.8) | 59 (70.2) | 14 (58.3) | 28 (75.7) | 7 (63.6) |
| Anti-EGFR agent | | | | | |
| Nituzumab | 35 (74.5) | 47 (56.0) | 19 (79.2) | 24 (64.9) | 7 (63.6) |
| Cetuximab | 12 (25.5) | 37 (44.0) | 5 (20.8) | 13 (35.1) | 4 (36.4) |
| Pathological histology | | | | | |
| Undifferentiated non-keratosis | 43 (91.5) | 80 (95.2) | 22 (91.6) | 32 (86.5) | 10 (90.9) |
| Others^†^ | 4 (8.5) | 4 (4.8) | 2 (8.4) | 5 (13.5) | 1 (9.1) |
| Recurrence/Metastasis sequence | | | | | |
| Synchronous | 35 (74.5) | 55 (65.5) | 6 (25.0) | 3 (8.1) | 1 (9.1) |
| Metachronous | 12 (25.5) | 29 (34.5) | 18 (75.0) | 34 (91.9) | 10 (90.9) |
| Karnofsky Performance Score (KPS) | | | | | |
| ＞ 80 | 40 (85.1) | 71 (84.5) | 21 (87.5) | 32 (86.5) | 9 (81.8) |
| ≤ 80 | 7 (14.9) | 13 (15.5) | 3 (12.5) | 5 (13.5) | 2 (18.2) |
| Baseline Epstein-Barr virus DNA level (copies/mL) | | | | | |
| ＜10E3 | 9 (19.1) | 5 (5.9) | 4 (16.7) | 7 (19.0) | 1 (9.1) |
| ≥10E3 and ＜10E4 | 8 (17.0) | 11 (13.1) | 3 (12.5) | 9 (24.3) | 1 (9.1) |
| ≥10E4 and ＜10E5 | 12 (25.5) | 24 (28.6) | 5 (20.8) | 12 (32.4) | 4 (36.4) |
| ≥10E5 | 11 (23.5) | 35 (41.7) | 9 (37.5) | 8 (21.6) | 4 (36.4) |
| Unknown | 7 (14.9) | 9 (10.7) | 3 (12.5) | 1 (2.7) | 1 (9.1) |

Footnote: †: Other pathological histology types contained non-keratosis, differentiated non-keratosis, squamous carcinoma, and unknown type. ‡: Other chemotherapy regimens included pemetrexed + cisplatin/nedaplatin, pemetrexed + gemcitabine, gemcitabine + capecitabine/S-1, gemcitabine + oxaliplatin, and gemcitabine + vincristine. Abbreviations: EGRF, epidermal growth factor receptor; TPF, taxane plus cisplatin/nedaplatin/carboplatin and fluorouracil; TP, taxane plus cisplatin/nedaplatin/carboplatin; PF, fluorouracil plus cisplatin/nedaplatin/carboplatin; GP, gemcitabine plus cisplatin/nedaplatin/carboplatin.
