# Supplementary material for: Anti‐epidermal growth factor receptor monoclonal antibody plus palliative chemotherapy as a first‐line treatment for recurrent or metastatic nasopharyngeal carcinoma
Source: Cancer Med. 2020 Jan 19;9(5):1721–32. doi: 10.1002/cam4.2838 (PMC7050081; doi:10.1002/cam4.2838)
Supplement: Supplementary file 2 [file CAM4-9-1721-s002.docx]

sTable 2 Common treatment-related adverse events in different chemotherapy regimens

| Adverse events | No (%) | Grade 1 (%) | Grade 2 (%) | Grade 3 (%) | Grade 4 (%) | Grade 3+4 (%) | All grade (%) |
| --- | --- | --- | --- | --- | --- | --- | --- |
| Leukopenia | | | | | | | |
| TPF | 4 (8.5) | 3 (6.4) | 21 (44.7) | 17 (36.2) | 2 (4.3) | 19 (40.5) | 43 (91.5) |
| TP | 21 (25.0) | 8 (9.5) | 14 (16.7) | 29 (34.5) | 12 (14.3) | 41 (48.8) | 63 (75.0) |
| PF | 0 (0.0) | 5 (20.8) | 10 (41.7) | 9 (37.5) | 0 (0.0) | 9 (37.5) | 24 (100.0) |
| GP | 4 (10.8) | 5 (13.5) | 13 (35.1) | 10 (27.0) | 5 (13.5) | 15 (40.5) | 33 (89.2) |
| Others^†^ | 3 (27.2) | 0 (0.0) | 4 (36.4) | 4 (36.4) | 0 (0.0) | 4 (36.4) | 8 (72.8) |
| Thrombocytopenia | | | | | | | |
| TPF | 28 (59.6) | 5 (10.6) | 9 (19.1) | 5 (10.6) | 0 (0.0) | 5 (10.6) | 19 (40.4) |
| TP | 64 (76.2) | 10 (11.9) | 6 (7.1) | 1 (1.2) | 3 (3.6) | 4 (4.8) | 20 (23.8) |
| PF | 13 (54.2) | 6 (25.0) | 3 (12.5) | 1 (4.2) | 1 (4.2) | 2 (8.4) | 11 (45.8) |
| GP | 9 (24.3) | 7 (18.9) | 9 (24.3) | 6 (16.2) | 6 (16.2) | 12 (32.4) | 28 (75.7) |
| Others^†^ | 6 (54.5) | 1 (9.1) | 4 (36.4) | 0 (0.0) | 0 (0.0) | 0 (0.0) | 5 (45.5) |
| Vomiting | | | | | | | |
| TPF | 24 (51.1) | 20 (42.6) | 3 (6.4) | 0 (0.0) | 0 (0.0) | 0 (0.0) | 23 (48.9) |
| TP | 58 (69.0) | 22 (26.2) | 3 (3.6) | 1 (1.2) | 0 (0.0) | 1 (1.2) | 26 (31.0) |
| PF | 5 (20.8) | 17 (70.8) | 1 (4.2) | 1 (4.2) | 0 (0.0) | 1 (4.2) | 19 (79.2) |
| GP | 27 (73.0) | 9 (24.3) | 1 (2.7) | 0 (0.0) | 0 (0.0) | 0 (0.0) | 10 (27.0) |
| Others^†^ | 10 (90.9) | 0 (0.0) | 1 (9.1) | 0 (0.0) | 0 (0.0) | 0 (0.0) | 1 (9.1) |
| Nausea | | | | | | | |
| TPF | 9 (19.1) | 28 (59.6) | 9 (19.1) | 1 (2.1) | 0 (0.0) | 1 (2.1) | 38 (80.9) |
| TP | 40 (47.6) | 37 (44.0) | 7 (8.3) | 0 (0.0) | 0 (0.0) | 0 (0.0) | 44 (52.4) |
| PF | 1 (4.2) | 20 (83.3) | 2 (8.3) | 1 (4.2) | 0 (0.0) | 1 (4.2) | 23 (95.8) |
| GP | 21 (56.8) | 14 (37.8) | 2 (5.4) | 0 (0.0) | 0 (0.0) | 0 (0.0) | 16 (43.2) |
| Others^†^ | 9 (81.8) | 2 (18.2) | 0 (0.0) | 0 (0.0) | 0 (0.0) | 0 (0.0) | 2 (18.2) |
| Mucosal inflammation | | | | | | | |
| TPF | 38 (80.9) | 4 (8.5) | 4 (8.5) | 1 (2.1) | 0 (0.0) | 1 (2.1) | 9 (19.1) |
| TP | 68 (81.0) | 8 (9.5) | 7 (8.3) | 1 (1.2) | 0 (0.0) | 1 (1.2) | 16 (19.0) |
| PF | 15 (62.5) | 6 (25.0) | 3 (12.5) | 0 (0.0) | 0 (0.0) | 0 (0.0) | 9 (37.5) |
| GP | 34 (91.9) | 3 (8.1) | 0 (0.0) | 0 (0.0) | 0 (0.0) | 0 (0.0) | 3 (8.1) |
| Others^†^ | 8 (72.7) | 3 (27.3) | 0 (0.0) | 0 (0.0) | 0 (0.0) | 0 (0.0) | 3 (27.3) |
| Decreased appetite | | | | | | | |
| TPF | 7 (14.9) | 34 (72.3) | 6 (12.8) | 0 (0.0) | 0 (0.0) | 0 (0.0) | 40 (85.1) |
| TP | 36 (42.9) | 42 (50.0) | 6 (7.1) | 0 (0.0) | 0 (0.0) | 0 (0.0) | 48 (57.1) |
| PF | 1 (4.2) | 22 (91.7) | 1 (4.2) | 0 (0.0) | 0 (0.0) | 0 (0.0) | 23 (95.8) |
| GP | 15 (40.5) | 20 (54.1) | 2 (5.4) | 0 (0.0) | 0 (0.0) | 0 (0.0) | 22 (59.5) |
| Others^†^ | 9 (81.8) | 2 (18.2) | 0 (0.0) | 0 (0.0) | 0 (0.0) | 0 (0.0) | 2 (18.2) |
| Diarrhea | | | | | | | |
| TPF | 42 (89.4) | 4 (8.5) | 0 (0.0) | 1 (2.1) | 0 (0.0) | 1 (2.1) | 5 (10.6) |
| TP | 70 (83.3) | 12 (14.3) | 1 (1.2) | 1 (1.2) | 0 (0.0) | 1 (1.2) | 14 (16.7) |
| PF | 17 (70.8) | 7 (29.2) | 0 (0.0) | 0 (0.0) | 0 (0.0) | 0 (0.0) | 7 (29.2) |
| GP | 35 (94.6) | 1 (2.7) | 1 (2.7) | 0 (0.0) | 0 (0.0) | 0 (0.0) | 2 (5.4) |
| Others^†^ | 11 (100.0) | 0 (0.0) | 0 (0.0) | 0 (0.0) | 0 (0.0) | 0 (0.0) | 0 (0.0) |
| Nephrotoxicity | | | | | | | |
| TPF | 36 (76.6) | 10 (21.3) | 1 (2.1) | 0 (0.0) | 0 (0.0) | 0 (0.0) | 11 (23.4) |
| TP | 74 (88.1) | 9 (10.7) | 1 (1.2) | 0 (0.0) | 0 (0.0) | 0 (0.0) | 10 (11.9) |
| PF | 14 (58.3) | 10 (41.7) | 0 (0.0) | 0 (0.0) | 0 (0.0) | 0 (0.0) | 10 (41.7) |
| GP | 29 (78.4) | 8 (21.6) | 0 (0.0) | 0 (0.0) | 0 (0.0) | 0 (0.0) | 8 (21.6) |
| Others^†^ | 10 (90.9) | 1 (9.1) | 0 (0.0) | 0 (0.0) | 0 (0.0) | 0 (0.0) | 1 (9.1) |
| Hypotension | | | | | | | |
| TPF | 44 (93.6) | 3 (6.4) | 0 (0.0) | 0 (0.0) | 0 (0.0) | 0 (0.0) | 3 (6.4) |
| TP | 67 (79.8) | 17 (20.2) | 0 (0.0) | 0 (0.0) | 0 (0.0) | 0 (0.0) | 17 (20.2) |
| PF | 21 (87.5) | 3 (12.5) | 0 (0.0) | 0 (0.0) | 0 (0.0) | 0 (0.0) | 3 (12.5) |
| GP | 30 (81.1) | 7 (18.9) | 0 (0.0) | 0 (0.0) | 0 (0.0) | 0 (0.0) | 7 (18.9) |
| Others^†^ | 7 (63.6) | 4 (36.4) | 0 (0.0) | 0 (0.0) | 0 (0.0) | 0 (0.0) | 4 (36.4) |
| Weight loss | | | | | | | |
| TPF | 31 (66.0) | 12 (25.5) | 4 (8.5) | 0 (0.0) | 0 (0.0) | 0 (0.0) | 16 (34.0) |
| TP | 62 (73.8) | 14 (16.7) | 8 (9.5) | 0 (0.0) | 0 (0.0) | 0 (0.0) | 22 (26.2) |
| PF | 11 (45.8) | 10 (41.7) | 3 (12.5) | 0 (0.0) | 0 (0.0) | 0 (0.0) | 13 (54.2) |
| GP | 29 (78.4) | 7 (18.9) | 1 (2.7) | 0 (0.0) | 0 (0.0) | 0 (0.0) | 8 (21.6) |
| Others^†^ | 9 (81.8) | 2 (18.2) | 0 (0.0) | 0 (0.0) | 0 (0.0) | 0 (0.0) | 2 (18.2) |
| Rash | | | | | | | |
| TPF | 42 (89.4) | 5 (10.6) | 0 (0.0) | 0 (0.0) | 0 (0.0) | 0 (0.0) | 5 (10.6) |
| TP | 71 (84.5) | 7 (8.3) | 4 (4.8) | 2 (2.4) | 0 (0.0) | 2 (2.4) | 13 (15.5) |
| PF | 21 (87.5) | 2 (8.3) | 0 (0.0) | 1 (4.2) | 0 (0.0) | 1 (4.2) | 3 (12.5) |
| GP | 32 (86.5) | 5 (13.5) | 0 (0.0) | 0 (0.0) | 0 (0.0) | 0 (0.0) | 5 (13.5) |
| Others^†^ | 10 (90.9) | 1 (9.1) | 0 (0.0) | 0 (0.0) | 0 (0.0) | 0 (0.0) | 1 (9.1) |
| Fever | | | | | | | |
| TPF | 28 (59.6) | 14 (29.8) | 5 (10.6) | 0 (0.0) | 0 (0.0) | 0 (0.0) | 19 (40.4) |
| TP | 60 (71.4) | 20 (23.8) | 4 (4.8) | 0 (0.0) | 0 (0.0) | 0 (0.0) | 24 (28.6) |
| PF | 19 (79.1) | 4 (16.7) | 1 (4.2) | 0 (0.0) | 0 (0.0) | 0 (0.0) | 5 (20.9) |
| GP | 26 (70.3) | 9 (24.3) | 2 (5.4) | 0 (0.0) | 0 (0.0) | 0 (0.0) | 11 (29.7) |
| Others^†^ | 10 (90.9) | 1 (9.1) | 0 (0.0) | 0 (0.0) | 0 (0.0) | 0 (0.0) | 1 (9.1) |
| ALT elevation | | | | | | | |
| TPF | 21 (44.7) | 22 (46.8) | 1 (2.1) | 3 (6.4) | 0 (0.0) | 3 (6.4) | 26 (55.3) |
| TP | 55 (65.5) | 24 (28.6) | 2 (2.4) | 3 (3.6) | 0 (0.0) | 3 (3.6) | 29 (34.5) |
| PF | 15 (62.5) | 7 (29.2) | 2 (8.3) | 0 (0.0) | 0 (0.0) | 0 (0.0) | 9 (37.5) |
| GP | 26 (70.3) | 9 (24.3) | 1 (2.7) | 1 (2.7) | 0 (0.0) | 1 (2.7) | 11 (29.7) |
| Others^†^ | 9 (81.8) | 2 (18.2) | 0 (0.0) | 0 (0.0) | 0 (0.0) | 0 (0.0) | 2 (18.2) |
| AST elavation | | | | | | | |
| TPF | 32 (68.1) | 11 (23.4) | 2 (4.3) | 2 (4.3) | 0 (0.0) | 2 (4.3) | 15 (31.9) |
| TP | 55 (65.5) | 24 (28.6) | 2 (2.4) | 3 (3.6) | 0 (0.0) | 3 (3.6) | 29 (34.5) |
| PF | 16 (66.7) | 8 (33.3) | 0 (0.0) | 0 (0.0) | 0 (0.0) | 0 (0.0) | 8 (33.3) |
| GP | 27 (73.0) | 9 (24.3) | 1 (2.7) | 0 (0.0) | 0 (0.0) | 0 (0.0) | 10 (27.0) |
| Others^†^ | 9 (81.8) | 2 (18.2) | 0 (0.0) | 0 (0.0) | 0 (0.0) | 0 (0.0) | 2 (18.2) |

Footnote: †: Other chemotherapy regimens included pemetrexed + cisplatin/nedaplatin, pemetrexed + gemcitabine, gemcitabine + capecitabine/S-1, gemcitabine + oxaliplatin, and gemcitabine + vincristine. Abbreviations: TPF, taxane plus cisplatin/nedaplatin/carboplatin and fluorouracil; TP, taxane plus cisplatin/nedaplatin/carboplatin; PF, fluorouracil plus cisplatin/nedaplatin/carboplatin; GP, gemcitabine plus cisplatin/nedaplatin/carboplatin. ALT, alanine aminotransferase; AST, aspartate aminotransferase.
